# Phyllodes Tumor: A Rare Cause of False-Positive Pregnancy Test Result

**DOI:** 10.7759/cureus.77071

**Published:** 2025-01-07

**Authors:** Rachel A Kracaw, Savannah Cotter, Ingmar N Bastian, Yingao Zhang, Jessica Grenvik, Kelly Blazek

**Affiliations:** 1 Obstetrics and Gynecology, Baylor College of Medicine, Houston, USA; 2 Gynecology Oncology, Cedars-Sinai Medical Center, Los Angeles, USA

**Keywords:** beta-human chorionic gonadotropin, breast mass, false-positive pregnancy test, phyllodes tumor, tumor marker

## Abstract

Phyllodes tumors (PT) are rare fibroepithelial neoplasms of the breast that are predominantly benign, but can exhibit malignant characteristics. Elevated beta-human chorionic gonadotropin (beta-hCG) levels, primarily associated with pregnancy and trophoblastic tumors, have been rarely reported in breast cancers and PT. We present a 28-year-old premenopausal female with a rapidly growing, painful mass in the right breast. Imaging and biopsy confirmed a malignant PT. Surprisingly, preoperative testing revealed a positive urine pregnancy test (UPT) and an elevated beta-hCG level (1,152.7 mlU/mL). After pregnancy was deemed unlikely, the patient proceeded with a right total mastectomy. Postoperatively, the beta-hCG level decreased, confirming tumor-related phenomenon. Pathology revealed malignant PT with liposarcomatous components and ductal carcinoma in situ (DCIS). The association between PT and elevated beta-hCG levels is exceptionally rare and poorly understood. This case highlights the importance of considering unusual presentations in PT patients.

## Introduction

Phyllodes tumors (PT) are rare fibroepithelial neoplasms that account for less than one percent of all breast tumors. They predominantly occur in women aged 30 to 50 years but can also affect younger and older individuals [1]. PT are usually benign, but approximately 20% exhibit malignant characteristics, including increased mitotic activity, stromal overgrowth, and infiltrative margins. The vast majority of PT are estrogen receptor (ER), progesterone receptor (PR), and human epidermal growth factor receptor 2 (HER2) negative [2,3]. The first line treatment of PT is complete surgical excision with the goal of negative margins. Adjuvant radiation is utilized in patients treated with wide local excision alone, and can be considered in patients with large tumors (over 10 cm) in the post-mastectomy setting. Adjuvant chemotherapy is controversial due to unclear evidence of efficacy, but sometimes utilized in the setting of metastatic disease [1,4].

Beta-human chorionic gonadotropin (beta-hCG) is a glycoprotein hormone produced by placental syncytiotrophoblasts and is primarily associated with pregnancy and trophoblastic tumors. However, beta-hCG may also be produced by other tissues such as testis, colon, liver, lung, and stomach [5]. As a result, elevated beta-hCG levels outside of pregnancy or trophoblastic tumors can occur in various malignancies, including testicular germ cell tumors, ovarian germ cell tumors, and some rare extragonadal neoplasms. Interestingly, lung tumors appear to be the most common beta-hCG producing non-gynecologic tumors in reproductive-age women [5]. Although elevated beta-hCG has been reported in breast cancers, and even in cases of PT, the incidence appears to be exceptionally rare and whether this association has clinical significance remains poorly understood [6,7]. Additionally, cases of PT have been reported in pregnancy, and it may be difficult to distinguish between early pregnancy or paraneoplastic syndrome [8]. We present a case of histology-confirmed malignant phyllodes tumor with abnormally elevated beta-hCG levels.

## Case presentation

This case report involves retrospective review of three or less individuals, and therefore an IRB review was not required. Informed written patient consent was obtained for publication of this case report.

**Figure 1:**
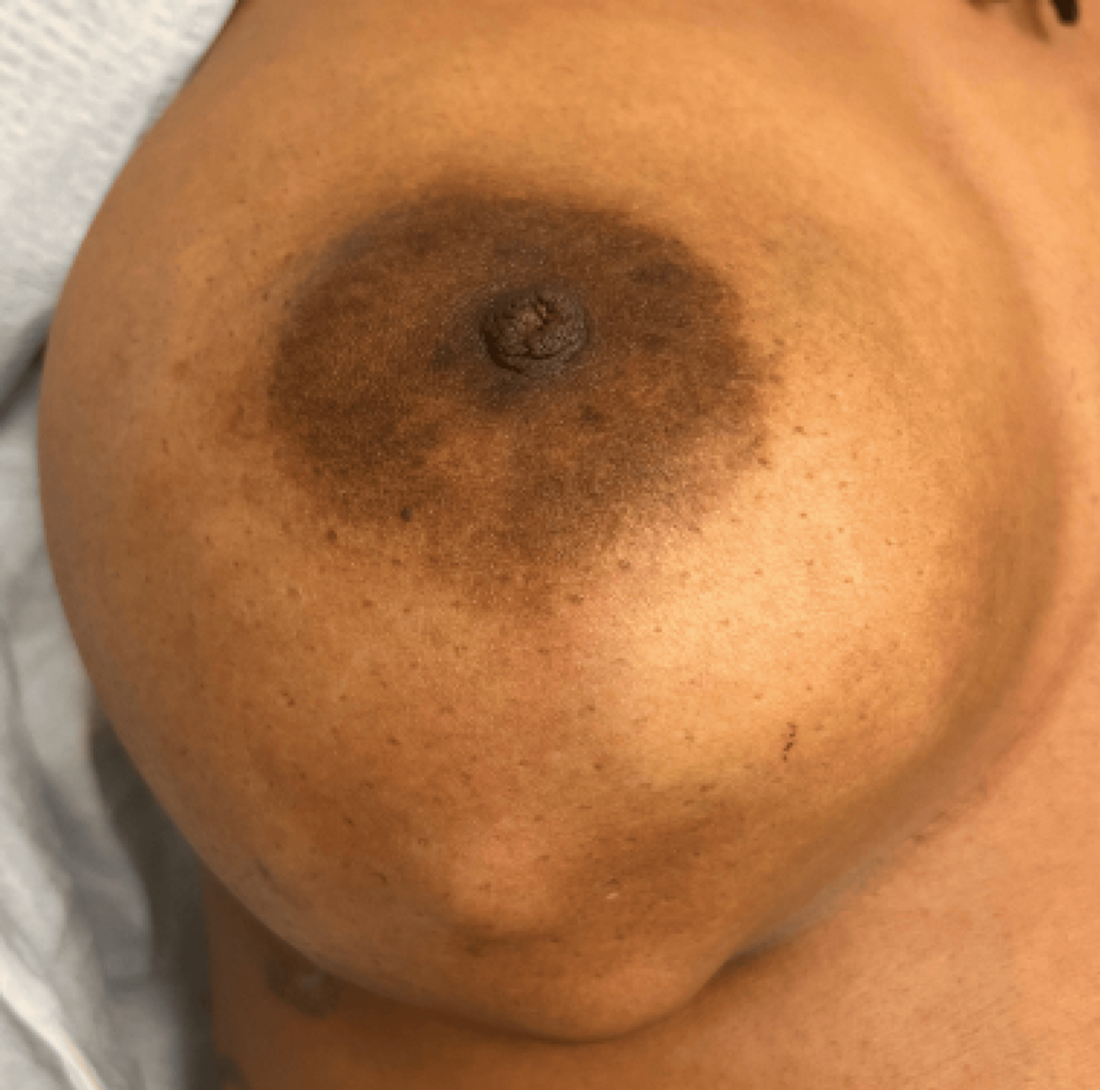
Breast mass in lower quadrant of breast with overlying skin changes

A 28-year-old premenopausal female presented to the emergency department with a painful, rapidly growing mass in her right breast. Past medical history was remarkable for fibrocystic breast changes and daily cigarette use. Her family history was positive for breast cancer in her grandmother. The patient was currently abstinent and not using contraception. Physical examination revealed a palpable and visibly firm, mobile mass in the lower quadrant of the breast, with overlying skin changes and mild erythema (Figure 1).

**Figure 2:**
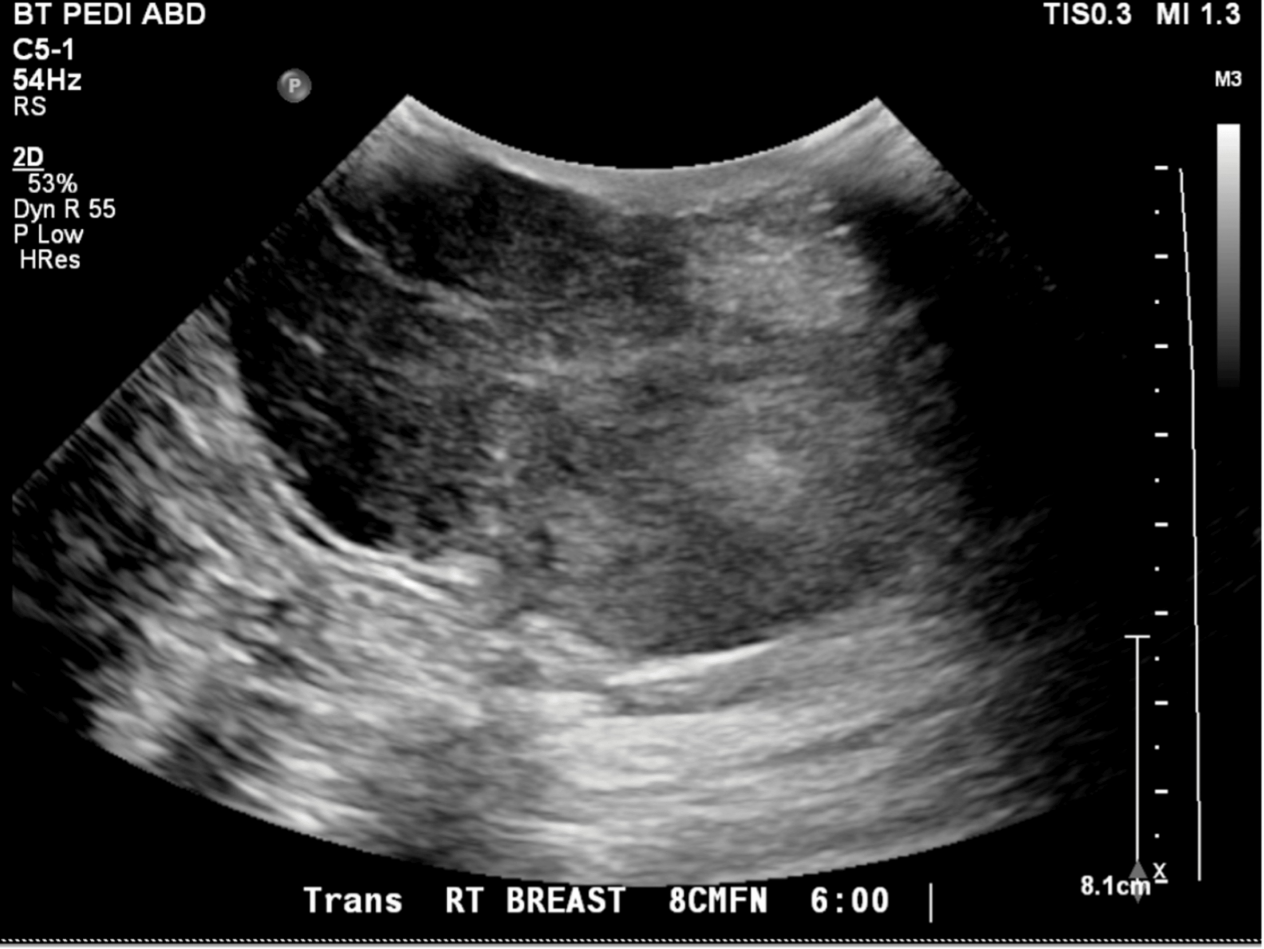
Breast ultrasound image (I) The image is showing a 10.1 x 5.4 x 7.5 cm partially solid mass with concern for possible malignancy.

**Figure 3:**
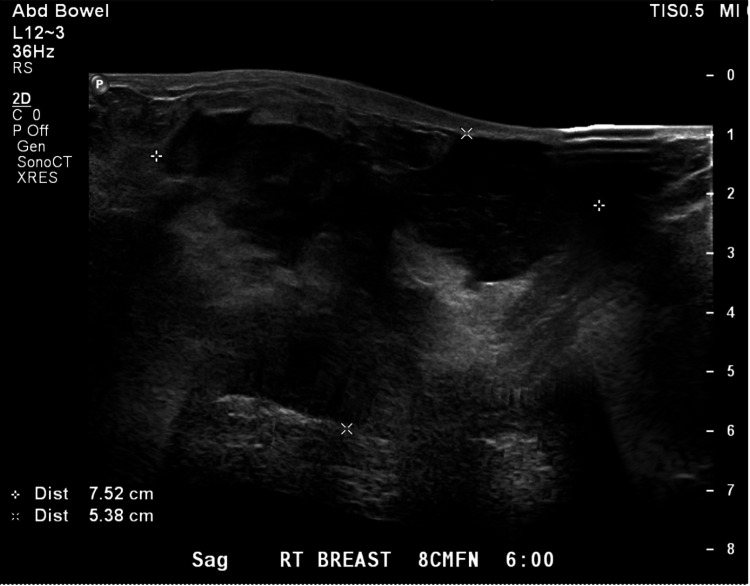
Breast ultrasound image (II) Breast ultrasound showing 10.1 x 5.4 x 7.5 cm partially solid mass with concern for possible malignancy.

The urine pregnancy test (UPT) was negative and other laboratory results were significant for an elevated erythrocyte sedimentation rate (ESR) of 61 mm/hr. Breast ultrasound showed a 10.1 x 5.4 x 7.5 cm partially solid mass with concern for possible malignancy (Figures 2, 3). Ultrasound-guided core needle biopsy showed a fibroepithelial lesion with stromal expansion, increased stromal cellularity, stromal nuclear atypia, and scattered mitotic figures consistent with a malignant PT.

**Figure 4:**
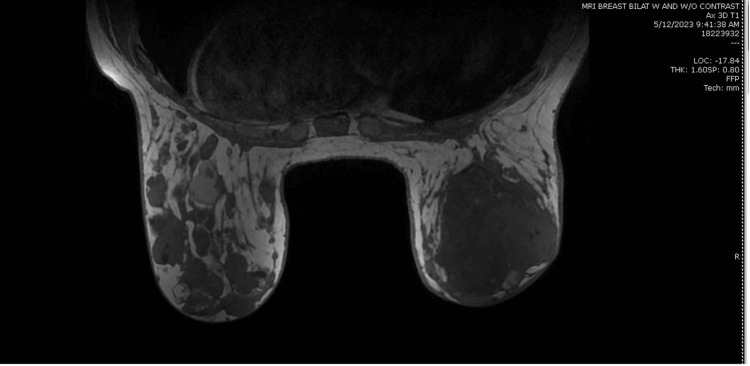
Breast MRI image The image is showing the right-sided lesion measuring 10.9 cm in greatest dimension with overlying skin thickening and enhancement.

MRI of the breast showed the right-sided lesion measuring 10.9 cm in greatest dimension with overlying skin thickening and enhancement (Figure 4). There was also a dominant 3.5 cm irregular mixed solid and cystic mass in the left breast, as well as >20 additional similarly enhancing smaller masses spread throughout the bilateral breasts. Bilateral level 1 axillary lymph nodes were enlarged to >2 cm without associated internal mammary adenopathy, which was indeterminate at the time of MRI. Additional biopsy of the left breast mass returned ductal hyperplasia and pseudoangiomatous stromal hyperplasia without atypia or malignancy. Further ultrasound evaluation during the left breast biopsy determined no significant axillary lymphadenopathy, and no additional biopsies were taken. After consultation with surgery and oncology teams, the patient elected for a right total mastectomy with delayed reconstruction.

On the day of surgery, required preoperative laboratory tests were significant for a positive UPT and the Obstetrics and Gynecology team was consulted. Serum beta-hCG confirmed positivity with a value of 1,152.7 mlU/mL.

The patient reported regular monthly menses, with her last cycle ending the day before her scheduled surgery, as well as abstinence since her diagnosis of malignancy. Bedside transabdominal ultrasound showed a normal-appearing uterus with a thin endometrial stripe and no intrauterine pregnancy. Bilateral adnexa were normal, appearing without masses. Pregnancy was unable to be ruled out given the serum beta-hCG under the discriminatory zone (3500 mIU/mL); however, it was deemed unlikely due to history and examination. Other diagnoses were considered, including paraneoplastic syndromes. After counseling and discussion, she elected to proceed with her surgery as scheduled.

Her surgery was uncomplicated. Surgical pathology confirmed a malignant PT with high-grade liposarcomatous components, as well as DCIS within the phyllodes tumor. There was atypical ductal hyperplasia noted in the breast tissue outside of the tumor, but all surgical margins were negative for malignancy. Postoperatively, repeat labs demonstrated a consistently decreasing beta-hCG of 48 mIU/mL and 24 mIU/mL on postoperative days 1 and 2, respectively. There was no abnormal vaginal bleeding or abdominopelvic pain in the immediate postoperative period, confirming the diagnosis of paraneoplastic syndrome.

Adjuvant radiation was recommended by radiation oncology due to the increased risk of local recurrence in patients with malignant phyllodes over 10 centimeters in the post-mastectomy setting. The patient completed adjuvant radiation to the breast six months after her surgery. Although pre-operative breast ultrasound noted no axillary lymphadenopathy, the patient presented with a painful mass in her right axilla seven months following surgery. A biopsy showed a malignant high-grade spindle cell lesion consistent with the recurrence of PT. Her beta-hCG at the time of diagnosis of recurrence was 116 mIU/mL.

The patient underwent an uncomplicated right axillary PT resection and lymph node dissection with surgical oncology nine months after her original surgery. Her beta-hCG on the day of surgery was 1486 mIU/mL. On postoperative day 6, beta-hCG had decreased to 35.7 mIU/mL. Surgical pathology confirmed a metastatic malignant PT involving axillary lymph nodes with negative margins. beta-hCG trends throughout diagnosis and treatment can be seen in Table 1.

**Table 1:** Beta-hCG trend throughout diagnosis and treatment POD: Postoperative Day; Beta-hCG: Beta-Human Chorionic Gonadotropin (Normal <5.0 mIU/mL in non-pregnant individuals).

Date	July 13, 2023	July 14, 2023	July 15, 2023	February 15, 2024	April 9, 2024	April 15, 2024
Treatment Day	Surgery #1	POD #1	POD #2	Recurrence Diagnosis	Surgery #2	POD #5
Beta-hCG (mIU/mL)	1,152.7	48.0	24.0	116.0	1,568.0	35.7

Following surgery for axillary recurrence of PT, the patient was diagnosed with multiple lung metastases on imaging. At the time of writing the case report, the patient was undergoing adjuvant chemotherapy for metastatic PT with adriamycin, ifosfamide, and mesna (AIM).

## Discussion

The association between PT and elevated beta-hCG levels is exceedingly rare, with only a handful of case reports published in the literature [6,7]. The exact underlying mechanism remains unclear. It is possible that this association represents an incidental finding rather than a direct relationship between the tumor and beta-hCG production. Additionally, PT in pregnancy has also been described in the literature, and pregnancy can be difficult to rule out with low levels of beta-hCG [8]. In our case, given the trend of beta-hCG throughout treatment progression, paraneoplastic beta-hCG secretion by the tumor seems the most likely mechanism. Our case describes a patient with PT and elevated beta-hCG that is younger than previous case reports, leading to a broad differential for elevated beta-hCG, including pregnancy.

The most common causes of false positive pregnancy tests are operator error in performing or interpreting the test, biochemical pregnancy (loss very soon after implantation or prior to US visualization), exogenous hCG administration secondary to infertility treatment or athletic performance, beta-hCG secreting tumors, pituitary HCG secretion in perimenopausal or postmenopausal women, interface with assay by anti-animal or anti-HCG antibodies or familial HCG syndrome [9]. Common tumors that secrete beta-hCG are lung cancers, ovarian germ cell tumors, gestational trophoblastic disease, benign or malignant teratomas, and very rare reports of phyllodes tumors or cervical squamous cell carcinomas [5,6,9,10]. It is important to consider non-traditional types of tumors when looking for causes of falsely elevated beta-hCG.

There have been a small number of case reports describing elevated beta-hCG with PT in the literature, and these two cases also describe an associated decrease in beta-hCG after resection of the tumor [6,7]. One case also reports increased levels of beta-hCG with recurrence of the tumor and associated decrease with continued treatment [7]. Our case also describes this phenomenon, with beta-hCG trending down in the immediate post-operative period and increasing with the recurrence of the disease. This trend supports the hypothesis of direct beta-hCG secretion by the tumor. This can also be useful in the care of patients with these rare tumors, as beta-hCG should be considered as a tumor marker throughout surveillance and treatment.

PT is known to exhibit diverse histological features and molecular alterations, including overexpression of various growth factors and hormone receptors. It is conceivable that molecular aberrations could influence the secretion of hormones or hormone-like substances, such as beta-hCG, albeit through unknown mechanisms. In fact, PT has been reported to be associated with other paraneoplastic syndromes, such as hypertrophic osteoarthropathy and hypoglycemia, due to IGF-2 production [11-13].

Our case describes a rare pathology of DCIS arising from the PT. It is unclear if this pathology contributes to the beta-hCG elevation, as there is little description of this phenomenon in the literature. DCIS and other breast cancers have been linked to elevated beta-hCG, and one study correlates beta-hCG level to tumor stage. However, the beta-hCG levels in this study are all < 5 mIU/mL [14]. Additionally, the small number of case reports describing elevated beta-hCG with PT does not describe a pathology similar to our case [6,7]. Therefore, the role of the DCIS component in our case is unclear, but it is unlikely the sole cause of beta-hCG elevation. 

Given the suspected malignant nature of the PT in this case and the absence of other evidence supporting trophoblastic disease or germ cell tumors, the patient was managed with total right mastectomy with complete tumor excision, followed by adjuvant radiation. Although the incidence of this finding appears to be extremely rare, surgical teams should be aware of its possibility to avoid delaying surgical resection due to misleading pre-operative testing. Additionally, beta-hCG should be considered for the surveillance of patients with this rare malignancy.

## Conclusions

We report a rare case of an elevated beta-hCG level in a patient with a malignant PT. The association between PT and elevated beta-hCG levels is exceptionally rare and poorly understood. Although the exact mechanism remains elusive, this case emphasizes the importance of considering unusual presentations and conducting thorough investigations to explore potential underlying causes in patients with PT. Further research is warranted to elucidate the possible association between PT and hormonal abnormalities such as elevated beta-hCG levels, as well as the use of beta-hCG levels during surveillance for these patients.
